# Mucosal Immunization Against Pertussis: Lessons From the Past and Perspectives

**DOI:** 10.3389/fimmu.2021.701285

**Published:** 2021-06-15

**Authors:** Violaine Dubois, Camille Locht

**Affiliations:** Univ. Lille, CNRS, Inserm, CHU Lille, Institut Pasteur de Lille, U1019 - UMR 8204 - CIIL - Center for Infection and Immunity of Lille, Lille, France

**Keywords:** pertussis, acellular vaccines, whole-cell vaccines, outer membrane vesicles, live attenuated vaccines

## Abstract

**Background:**

Current vaccination strategies against pertussis are sub-optimal. Optimal protection against *Bordetella pertussis*, the causative agent of pertussis, likely requires mucosal immunity. Current pertussis vaccines consist of inactivated whole *B. pertussis* cells or purified antigens thereof, combined with diphtheria and tetanus toxoids. Although they are highly protective against severe pertussis disease, they fail to elicit mucosal immunity. Compared to natural infection, immune responses following immunization are short-lived and fail to prevent bacterial colonization of the upper respiratory tract. To overcome these shortcomings, efforts have been made for decades, and continue to be made, toward the development of mucosal vaccines against pertussis.

**Objectives:**

In this review we systematically analyzed published literature on protection conferred by mucosal immunization against pertussis. Immune responses mounted by these vaccines are summarized.

**Method:**

The PubMed Library database was searched for published studies on mucosal pertussis vaccines. Eligibility criteria included mucosal administration and the evaluation of at least one outcome related to efficacy, immunogenicity and safety.

**Results:**

While over 349 publications were identified by the search, only 63 studies met the eligibility criteria. All eligible studies are included here. Initial attempts of mucosal whole-cell vaccine administration in humans provided promising results, but were not followed up. More recently, diverse vaccination strategies have been tested, including non-replicating and replicating vaccine candidates given by three different mucosal routes: orally, nasally or rectally. Several adjuvants and particulate formulations were tested to enhance the efficacy of non-replicating vaccines administered mucosally. Most novel vaccine candidates were only tested in animal models, mainly mice. Only one novel mucosal vaccine candidate was tested in baboons and in human trials.

**Conclusion:**

Three vaccination strategies drew our attention, as they provided protective and durable immunity in the respiratory tract, including the upper respiratory tract: acellular vaccines adjuvanted with lipopeptide LP1569 and c-di-GMP, outer membrane vesicles and the live attenuated BPZE1 vaccine. Among all experimental vaccines, BPZE1 is the only one that has advanced into clinical development.

## Introduction

Pertussis is a highly contagious and life-threatening respiratory disease, mainly caused by *Bordetella pertussis*. In its severe forms, the disease manifests as pronounced leukocytosis, pulmonary hypertension and eventually death. The recent resurgence of pertussis in vaccinated populations illustrates the limits of current pertussis vaccination programs. Although parenterally delivered pertussis vaccines confer high-level protection against the disease, especially against severe pertussis, they do not prevent nasal carriage and transmission of *B. pertussis* (1–4). In fact, nasopharyngeal carriage of *B. pertussis* in hosts that received acellular pertussis vaccines (aPV) may even be prolonged (1, 3, 4), and thereby augment continuous spread of the bacteria by transmission, which may have been a major contributor to the current resurgence of the disease (5).

Reducing nasal carriage by immunization may therefore be important to lower the risk of exposure and lessen transmission, especially to unvaccinated individuals. Prolonged immunity is also an important goal for novel pertussis vaccines, as rapid waning of immunity is a major issue of current aPV (6). Naturally occurring *B. pertussis* infection has been shown to confer long-lasting protection against subsequent infection, although even infection-induced immunity is usually not life-long (7). Nevertheless, prolonged immunity through infection may reflect the induction of persistent mucosal immune memory, which can be rapidly recalled at the respiratory mucosa upon re-exposure. *B. pertussis* infections induce strong local secretory antibody and Th17-type cellular immune responses that are protective against *B. pertussis* infection (8, 9). These types of immune responses are not efficiently induced by parenteral delivery of current pertussis vaccines.

Considering the importance in providing durable and sterilizing immunity at the respiratory mucosal sites (10), the aim of this review is to provide an overview of mucosal vaccines against pertussis, from mucosal administration of the first generation, whole-cell pertussis vaccines (wPV) over adjuvanted aPV to the development of novel, nasally delivered outer membrane vesicles (OMV) and live attenuated vaccines.

## Methods

### Literature Search and Data Extraction

**Figure 1:**
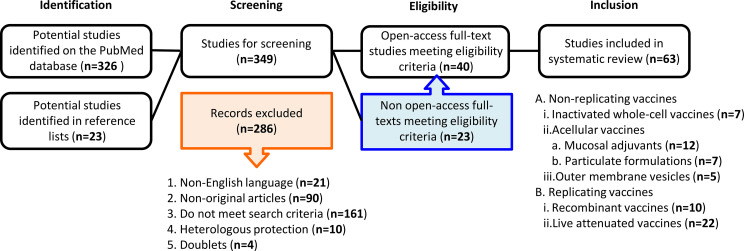
A flowchart of the methodology used to select the relevant publications. Among the initial total of 349 studies identified duplicates and articles providing corrections were excluded for the screening. At screening 286 articles were excluded, as they did not fulfill our eligibility criteria. Among the remaining 63 articles, 40 were open access, while 23 were not open access. All 63 articles were included in this review.

A systematic literature search was performed by both investigators independently. A comprehensive literature search of the PubMed Library database was conducted to identify articles published until February 2021. The key search terms were “pertussis mucosal vaccines”, “oral”, “nasal” and “rectal” to collect as many publications on mucosal immunization against pertussis as possible. Non-English publications were excluded. Reference lists of included studies were also searched for potentially relevant publications (snowball method). Data extraction was performed based on the predefined eligibility criteria. A flowchart summarizing the methodology is shown in **Figure 1**. The preparation of this review was guided by the PRISMA-P 2015 guideline (11).

### Eligibility Criteria

Non-English language publications and non-original articles (e.g. Review articles, recommendations, WHO reports, meeting reports) were excluded. The selection criteria for studies included mucosal administration of the vaccines and the evaluation of at least one outcome related to efficacy, immunogenicity and safety of the vaccines. Review articles were excluded.

### Data Synthesis and Analysis

Data are presented from oldest to the most recent in a manner that brings out the rational way forward to improve vaccines. Over the years, new vaccination strategies arose, and this review has been divided into non-replicating [inactivated wPV (n=7), aPV (n=19) and OMV (n=5)] and replicating [recombinant (n=10) and live attenuated (n=22)] vaccines (**Table 1**). When available, effects of the immunization route were compared.

**Table 1:** Main characteristics of mucosal vaccines against pertussis.

	Vaccine type		Model	Route	Immune response	Protection	Ref
**Non-replicating vaccines**	**Inactivated whole-cell vaccines (n=7)**						
		wPV*	Human	Aerosol	Specific mucosal IgA; no serum IgG	ND	(12)
		wPV	Human	Oral	Agglutinating salivary antibodies; low specific IgG in sera; cellular responses	Lower disease incidence the first year following immunization	(13)
		wPV	Human	Nasal	Specific mucosal responses in the nasal fluids (neglectable in the saliva); substantial serum antibodies	ND	(14)
		wPV + CT	Human	Nasal or rectal	Low systemic or mucosal responses following rectal immunization Specific IgA and IgG in serum, saliva and feces following nasal administration	Against nasopharyngeal colonization	(15)
		DTwP	Mouse	Oral	Specific serum IgG and IgA in saliva and intestinal secretion	ND	(16)
		wPV	Human	Nasal	Systemic cellular responses (Th1-type)		(17)
		wPV + curdlan	Mouse	Nasal	Th17 cells in the spleen; reduction of pro-inflammatory responses	Against colonization in the upper and lower respiratory tract; reduced leukocytosis	(18)
	**Acellular vaccines + mucosal adjuvants (n=12)**						
		aPV + LT	Mouse	Nasal	Th1 and Th2 cells in the spleen and lymph nodes; local and systemic specific antibody responses	Against lung colonization	(19)
		DTwP or PRN + LT	Neonatal mice	Nasal	T- and B-cell responses, poor serum antibody production	Against lung colonization	(20)
		PRN or FHA	Mouse	Nasal	Low antibody-secreting cells in lungs but strong priming	Against lung colonization	(21)
		PT and FHA + CTB	Mouse	Nasal	Specific systemic antibody responses; no mucosal antibodies	Against lung colonization	(22)
		PT + CTB	Mouse	Nasal	Mucosal IgA and systemic antibodies with anti-PT activity	Against lung colonization	(23)
		Fim2 + CTB	Mouse	Nasal	Th1 and Th2 cells; specific serum IgG and specific IgG and IgA in bronchoalveolar lavages	Against lung colonization	(24)
		DTwP + onjisaponin	Mouse	Nasal	Specific antibodies in the serum and in nasal washes	ND	(25)
		PT, FHA, PRN + BLP	Mouse	Nasal	Anti-PT and anti-FHA in the nasal washes	Against lung colonization	(26)
		DTaP + curdlan	Mouse	Nasal	Anti-PT and anti-FHA IgG in serum and lung IgA; Th17 response and neutrophilia; reduction of pulmonary pro-inflammatory cytokines	Against colonization of the upper and lower respiratory tract	(27)
		aPV + curdlan	Mouse	Nasal	Long-lasting serum and lung antibodies	Long-lasting against colonization in lower and upper respiratory tract; reduced inflammation	(28)
		PT, FHA, Prn + CpG ± alum	Mouse	Nasal	Specific antibodies in the serum and in the lungs; Th1 profile and activation of macrophages	Against lung colonization	(29)
		aPV + LP-GMP	Mouse	Nasal	Th17 response and memory T cells, reduction of Th2 responses	Long-lasting protection against nasal colonization	(30)
	**Acellular vaccines in particulate formulations (n=7)**						
		LPS or outer membrane proteins + liposomes	Mouse	Oral or nasal	Short-lived antibodies in lung washes; specific IgG, no IgA	ND	(31)
		PT in poly[di(sodium carboxylatophenoxy)phosphazene] microparticles	Mouse	Nasal	Specific systemic and mucosal antibodies; long-term systemic, lung and nasal memory B cells; mixed Th1/Th2 responses	Against lung colonization	(32)
		FHA, PT or PRN in poly-lactide-co-glycolide microspheres	Mouse	Nasal	Specific antibodies in the serum and in bronchoalveolar lavages	Against lung colonization	(33)
		FHA + PT in poly-lactide-co-glycolide microspheres	Mouse	Oral	Specific serum and mucosal antibodies; specific Th1 and Th2 responses	Against lung colonization	(34)
		Fimbria in poly-lactide-co-glycolide microsphere	Mouse	Oral	Specific systemic and local antibodies	Against colonization of the lungs and the trachea	(35)
		FHA + PT with N-trimethyl chitosan nanoparticles	Mouse	Nasal	Anti-PT and anti-FHA IgG and IgA in sera and nasal washes, respectively; Th2, Th1 and Th17 cellular responses	ND	(36)
		FHA + PT in chitosan	Mouse	Nasal	Enhanced anti-PT and anti-FHA antibodies in the nasal washes and anti-FHA IgG in serum	ND	(37)
	**OMV (n=5)**						
		OMV	Mouse	Nasal	Inflammatory chemokine CCL-20 and IL-6 and TNF-α cytokines in the first hours post-immunization	Against lung colonization	(38)
		OMV	Mouse	Nasal	Specific mucosal and systemic antibodies; memory B cells in lungs and spleen; Th1 and Th17 responses in lung and mixed Th1, Th2 and Th17 responses in the spleen	Against colonization of the upper and lower respiratory tract	(39)
		OMV*Bp*PagL	Mouse	Nasal	Reduction of inflammatory cytokines IL-6 and IL-1β	Against lung colonization	(40)
		OMV	Mouse	Pulmonary	Mucosal IgA and systemic IgG and IgG-secreting memory B cells; Th17 cells and reduction of Th2 cytokines and of IL-10 in serum	Against colonization of the upper and lower respiratory tract	(41)
		Spray-dried OMV	Mouse	Pulmonary	Mucosal IgA; broad systemic IgG; mixed Th1/Th17 responses	Against colonization in lower and upper respiratory tract	(42)
**Replicating vaccines**	**Recombinant vaccines (n=10)**						
		*S. dublin ΔaroA* + FHA	Mouse	Oral	Anti-FHA IgA in serum and gut washes; short-lived antibody responses	ND	(43)
		*S. typhimurium* Δ*aroA*Δ*aroD* + PRN	Mouse	Oral	Specific Th1-type response; no specific antibodies	ND	(44)
		*S. typhimurium* Δ*aroA* + FHA or *E. coli* + FHA	Mouse	Oral	Specific serum IgG + IgG and IgA in the lungs	ND	(45)
		*S. typhimurium* Δ*aroA* + PTS1, *S. typhimurium* Ty21a + PTS1 or *E. coli* + PTS1	Mouse	Oral	Specific serum IgG + IgG and IgA in the lungs	ND	(46)
		*S. typhimurium* Δ*aroA* + PT-S1-S5	Mouse	Oral	Specific serum IgG	No protection against intracerebral challenge	(47)
		*S. typhi* CVD 908 + PTS1	Mouse	Oral or Nasal	Neutralizing anti-PT antibodies after nasal vaccination	ND	(48)
		*V. cholerae* IEM101 + Tcf	Mouse	Nasal	No detectable antibodies	Against tracheal, but not lung colonization	(49)
		*L. lactis* + PTS1 + FHA type I immunodominant domain	Mouse	Oral and nasal	Specific serum IgG; specific IgA in lung and feces; nasal immunization induces a strong Th1 response	ND	(50)
		*S. gordonii* + PTS1	Mouse	Oral	Specific IgA in saliva; no serum antibodies	ND	(51)
		*S. mutans* + PTS1	Mouse	Oral	Specific mucosal IgA	Against lung colonization	(52)
	**Live attenuated *Bordetella* strains (n=22)**						
		*B. bronchiseptica* lacking ACT and the type III secretion system	Mouse	Nasal	Induction of Th1 response and reduction of IL-10 compared to the parental strain	Against colonization of the lower respiratory tract; reduced lung pathology	(53)
		*B. bronchiseptica* lacking ACT, FHA, PRN, Fim and BipA	Mouse	Nasal	Low serum antibody titers	Against colonization of the upper and lower respiratory tract; reduced lung pathology	(54)
		*B. pertussis* Δ*aroA*	Mouse	Respiratory	Serum IgG and IgGA	Against lung colonization	(55)
		*B. pertussis* Δ*aroQ*	Mouse	Nasal	Humoral and cellular responses (Th1 and Th2)	Against lung colonization	(56)
		*B. pertussis* Δ*ptx*	Mouse	Nasal	Serum IgG against FHA	Against lung colonization	(57)
		*B. pertussis* Δ*ptx*	Mouse	Nasal	Specific genital IgA and IgG	ND	(58)
		*B. pertussis* BPZE1	Adult and infant mice	Nasal	Antigen-specific antibodies and Th1 responses	Against lung colonization; reduced lung inflammation	(59)
		*B. pertussis* BPZE1	Mouse	Nasal	Dose-dependent antigen-specific serum antibodies, specific IgG and IgA in bronchoalveolar lavage fluids Dose-dependent Th1 responses	Against lung colonization in a dose-dependent manner; reduced lung inflammation	(60)
		*B. pertussis* BPZE1	Mouse	Nasal	Long-term specific serum IgG; IFN-γ-producing T cells	Against lung colonization	(61)
		*B. pertussis* BPZE1	Mouse	Aerosol	Long-lasting serum antibodies	Long-lasting against lung colonization	(62)
		*B. pertussis* BPZE1	Mouse	Nasal	IL-10, IL-17, IFN-γ and IL-2 following stimulation with FHA; induction of IL-17 and IFN-γ following stimulation with PTX	Against lung colonization	(63)
		*B. pertussis* BPZE1	Mouse	Nasal	IL-17-dependent secretory IgA in nasal washes and Th17 and Th1 T_RM_ cells in the nose	Against colonization of the lower and upper respiratory tract.	(64)
		*B. pertussis* BPZE1	Mouse	Nasal	Maintenance of Th1/Th17 responses after aPV boosting	Against lung colonization	(65)
		*B. pertussis* BPZE1	Adult and neonatal mice	Aerosol	Specific IgG2a and Th1 responses	Against lung inflammatory pathology	(66)
		*B. pertussis* BPZE1	Mouse	Nasal	ND	Early protection against lung colonization	(67)
		*B. pertussis* BPZE1	Baboon	Tracheal and nasal	Specific serum IgG and IgA	Against nasopharyngeal colonization; against leukocytosis	(68)
		*B. pertussis* BPZE1	Human	Nasal	Specific serum IgG	ND	(69)
		*B. pertussis* BPZE1	Human	Nasal	Specific serum IgG and IgA	ND	(70)
		*B. pertussis* BPZE1	Human	Nasal	Specific B cell responses	ND	(71)
		*B. pertussis* BPZE1	Human	Nasal	Specific T cell responses and broad serum antibody responses	ND	(72)
		*B. pertussis* BPZE1	Mouse	Nasal	Specific serum IgG	Against colonization in lower and upper respiratory tract in the presence of pre-existing anti-PRN antibodies	(73)
		*B. pertussis* BPZE1	Mouse	Nasal	Anti-Fim2 and anti-Fim3 serum antibodies	Against lung colonization by Fim3 only producing *B. pertussis*	(74)

ACT, adenylate cyclase toxin; aPV, acellular pertussis vaccine; BLP, bacterium-like particles; CT, cholera toxin; CTB, cholera toxin B subunit; DTaP, diphtheria-tetanus-acellular pertussis vaccine; DTwP, diphtheria-tetanus-whole-cell pertussis vaccine; wPV, whole-cell pertussis vaccine; FHA, filamentous haemagglutinin; Fim, fimbriae; LPS, lipopolysaccharide; LT, E. coli heat labile toxin; ND, not determined; OMV, outer membrane vesicles; OMV BpPagL, outer membrane vesicle from recombinant B. pertussis producing PagL; PRN, pertactin; PT, pertussis toxin; PTS1, pertussis toxin S1 subunit; Tcf, tracheal colonization factor; T_RM_, tissue-resident memory T cells.

## Results

### Non-Replicating Vaccines

Non-replicating vaccines include wPV, aPV containing a limited number of purified antigens and, more recently, *B. pertussis*-derived OMV. Non-replicating wPV were the first to be developed and used in humans since the beginning of the 20^th^ century. As injectable vaccines, they have been highly effective in preventing childhood deaths due to pertussis. However, frequent adverse events following immunization and lot inconsistencies have led to the development of aPV, which, with an improved safety profile, also provided strong protection as injectable vaccines. Mucosal administrations have been explored in an attempt to improve their efficacy.

#### Inactivated Whole-Cell Vaccines

The wPV are suspensions of entire *B. pertussis* organisms that have been inactivated, usually by formalin or heat treatment. Most wPV are available in combination with diphtheria (D) and tetanus (T) toxoids as DTwP. These vaccines prevent the disease in immunized individuals and are relatively inexpensive. They have been licensed for routine vaccination of infants since the mid-1940s.

Upon injection they induce specific systemic antibodies important to prevent pertussis disease (75, 76), but fail to induce mucosal antibodies that may be important to prevent colonization by and transmission of the pathogen (64). Few studies have evaluated antibody responses after mucosal administration of DTwP in human volunteers. In 1975, G. Thomas showed that respiratory administration of DTwP induces specific mucosal IgA, unlike immunization *via* the intramuscular route (12). However, respiratory immunization failed to induce serum IgG. Ten years later, oral administration of five very high doses of wPV to newborn babies was shown to induce salivary antibodies able to agglutinate the *Bordetella* organisms, as well as specific systemic IgG and cellular immune responses (13). Interestingly, these systemic immune responses occurred significantly earlier in the orally than in the parenterally vaccinated newborns, and anti-pertussis antibodies in saliva were not induced in the parenterally vaccinated Infants. However, the systemic antibody responses following mucosal immunization with wPV were rather low. Later, nasal administration of 4 doses of a wPV suspension consisting of 250 µg of protein at weekly intervals was shown to induce specific mucosal and substantial systemic antibody responses in human volunteers (14). While IgA to *B. pertussis* lysates was induced in nasal fluids of all vaccinees, the levels were negligeable in saliva.

Berstad et al. (15) attempted to identify formulations that optimize mucosal immunization with wPV in a mouse model by using alternative mucosal delivery routes or by using cholera toxin (CT), a well-known potent mucosal adjuvant. Mucosal immunization by rectal delivery did not result in any substantial systemic or mucosal antibody responses. While nasal immunization of mice with wPV gave rise to specific IgA and IgG in serum, as well as IgA in saliva and feces, the addition of CT curiously inhibited the induction of IgA in both serum and saliva of the mice. DTwP was also encapsulated using water-soluble chitosan and found to elicit *B. pertussis*-specific serum IgG, as well as IgA in saliva and intestinal secretions of mice after two oral administrations (16). A comparison with non-encapsulated DTwP was not reported in this study, and neither of these studies reported on the protective capacities of these formulations.

T-cell responses play also a role in protection against pertussis. When administered parenterally, wPV induced Th1-type immune responses, which are required for protection against colonization of the respiratory tract. Such T cell responses were also induced in human volunteers intranasally immunized with repeated 250 µg doses of wPV, as evidenced by antigen-specific proliferative T cell responses in the blood (17). In addition to Th1-type immune responses, Th17-type responses are also important for protection against pertussis, and are essential for prevention of nasal colonization (4). Recently, innate and adaptive immune responses to nasally administered wPV in conjunction with curdlan have been examined in a murine model of pertussis (18). Curdlan is a polysaccharide that forms a gel-like substance and is capable of inducing a strong Th17 response. Intranasal wPV immunization in this formulation reduced bacterial colonization in the upper and lower respiratory tract following *B. pertussis* challenge. It also reduced leukocytosis in the immunized mice and decreased the production of several pro-inflammatory cytokines upon challenge, while strongly inducing IL-17 one day post-challenge, as well as Th17 cells in the mouse spleens one week post-challenge. However, the induction of specific memory T cells was not examined. It has not yet been investigated whether mucosal administration of wPV together with curdlan elicits long-lasting protective immune responses, as would be desired for novel vaccination strategies.

Although clinical studies have shown that mucosally administered wPV are well tolerated in infants (12, 13), superiority of mucosally compared to parenterally administered wPV for prevention of pertussis disease or *B. pertussis* infection has not been demonstrated.

#### Acellular Vaccines

aPV have replaced wPV in most industrialized countries because of improved safety profiles. The first aPV, developed in Japan by Sato et al., contained chemically detoxified pertussis toxin (PT) and filamentous haemagglutinin (FHA) and was shown to be effective in the prevention of disease (77). Subsequently, pertactin (PRN) and serotype 2 (Fim2) and serotype 3 (Fim3) fimbriae were added to some aPV, which increased the potency of the vaccines (78). Today, most aPV are also combined with diphtheria and tetanus toxoids and are referred to as DTaP.

When administered parenterally to mice, aPV induce strong humoral responses but weak cellular Th1 and Th17 immune responses (79). Parenteral aPV immunization also fails to induce the mucosal immune responses (64). Therefore, in recent years work has been focused on the development of mucosally administered aPV. Unlike wPV that have intrinsic adjuvant properties when given nasally, most purified protein antigens are poorly immunogenic when ingested or inhaled and require the co-administration of potent mucosal adjuvants. The development of effective mucosal vaccines has long been hampered by the lack of appropriate and safe delivery systems and adjuvants to enhance these immune responses following mucosal immunization.

Licensed aPV contain the pro-Th2 aluminum salts, which is suboptimal for protection against *B. pertussis* infection. Adjuvants inducing more balanced T-helper profiles or Th1/Th17-type responses are needed for improved protection against infection. Different approaches have been taken to improve the immunogenicity of aPV, which include the use of microbial derivatives and particulate adjuvants. These experimental vaccines have been tested in mouse models, but have not yet been evaluated in humans.

##### Mucosal Adjuvants

CT and *Escherichia coli* heat labile toxin (LT) are among the most potent mucosal adjuvants. When delivered intranasally together with aPV, a non-toxic mutant form of LT enhanced both Th1 and Th2 T cell responses and provided equivalent protection to alum-absorbed aPV administered parenterally in a mouse model of pulmonary infection (19). However, protection was less strong than after parenterally administration of wPV. A subsequent study showed that one µg PRN as a single antigen combined with LT delivered twice intranasally to neonatal mice was sufficient to protect them from lung colonization by *B. pertussis*, although no serum antibody response to PRN could be detected (20). When larger doses of PRN or FHA (12 µg administered twice) were given intranasally to adult mice, the co-administration of LT was not required to protect the mice against lung colonization (21).

In a different study, co-administration of commercial aPV with CT subunit B (CTB) enhanced serum IgG and IgA responses in adult mice but also induced specific serum IgE to PT, which may be a safety concern (22). Subsequent studies have evaluated protection conferred by *B. pertussis* antigens genetically fused to recombinant CTB, such as the S1 subunit of PT and Fim2. The chimeric PT-CTB vaccine elicited specific IgA in the mucosa, as well as IgA and IgG in the serum after intranasal immunization, and the immune serum neutralized PT activity *in vitro* (23). The chimera reduced lung colonization by *B. pertussis*. However, cellular immune responses and nasal carriage of challenge bacteria in the vaccinated animals were not examined. Intranasal immunization with the Fim2-CTB chimera enhanced protection against lung colonization compared to intraperitoneal immunization and resulted in a mixed Th1/Th2 response in mice (24). Although intranasal vaccination with Fim2 fused to CTB resulted in enhanced lung clearance compared to vaccination with non-fused recombinant Fim2, it did not enhance clearance over intranasal vaccination with native Fim2 purified from *B. pertussis*. Since intranasal delivery of Fim2 itself was sufficient to generate antibody responses in serum and mucosal sites, the use of CTB as an adjuvant has been questioned. In addition, the use of CTB as a mucosal adjuvant for nasal delivery in humans is problematic, as its binding to gangliosides on nerve cells following intranasal administration has been reported (80), and this may lead to Bell’s palsy.

As a safer alternative to CTB, onjisaponin, extracted from the root of *Polygala tenuifolia*, was shown to provide as much adjuvanticity as CTB towards PT, diphtheria toxoid and tetanus toxoid upon intranasal delivery in mice (25). Other alternatives as safe mucosal adjuvants are bacterium-like delivery particles (BLP), composed of non-living particles of lactic acid bacteria (LAB), such as *Lactococcus lactis*. They consist of the bacterial peptidoglycan matrix obtained by treatment with hot acid. BLP were shown to enhance antibody responses when combined with inactivated PT, FHA and PRN and given intranasally to mice (26). Unlike alum-adjuvanted antigens, the use of BLP also led to substantial induction of anti-PT and anti-FHA IgA in the nasal washes. Furthermore, nasal immunization with BLP-loaded antigens conferred better protection against lung colonization than nasal immunization with alum-adjuvanted antigens given either parenterally or nasally. However, the ability of the BLP or of onjisaponin to enhance cellular immunity was not examined.

Boehm et al. showed recently that the pro-Th17 microbial derivative curdlan facilitates aPV localization to the upper respiratory tract (27). Intranasal immunization with this formulation was as effective as parenteral immunization in eliciting antibody responses against FHA and PT, while it induced less pulmonary pro-inflammatory cytokines. The use of curdlan compensated for IL-17 suppression by aPV and was associated with an increase in blood neutrophils. However, the frequency of neutrophils in the infected lungs was inferior compared to that of mice that had received wPV parenterally. Neither the frequency of CD4^+^ resident memory T (T_RM_) cells in the lungs, nor IgA levels in lung lavages were enhanced by the addition of curdlan. Instead, it appeared to suppress IgA responses in the nasal secretions, which, however, did not impair protection against nasal colonization. Importantly, compared to non-vaccinated controls, a significant reduction in bacterial burden in lungs, trachea and nasal tissues could still be observed 6 months after two intranasal administrations of curdlan-formulated aPV (28). This reduction in bacterial burden was correlated with the persistence of *B. pertussis*-specific antibody levels in lung and serum. Surprisingly, this was also seen with non-adjuvanted aPV or aPV combined with alum.

Another way to balance the immune responses induced by aPV is to add Toll-like receptor (TLR)-agonists to the vaccines, such as CpG derivatives. Oligonucleotides containing immunostimulatory CpG motifs (CpG ODN) interact with TLR-9, which initiates a cascade of events resulting in Th1 type cytokine and chemokine induction. Asokanathan et al. assessed CpG as a mucosal adjuvant for PT, FHA and PRN, alone or in combination with alum (29). The use of CpG ODN enhanced serum and lung antibody titers against these antigens, and in particular against PRN. It substantially enhanced nitric oxide synthase activity of macrophages compared to alum and shifted the T cell responses towards a Th1 profile. CpG ODN-formulated aPV protected against lung colonization at similar levels as alum-adjuvanted aPV, but co-administration of alum and CpG ODN substantially enhanced protection. Therefore, the alum-CpG ODN combination may be an attractive formulation to protect the lower respiratory tract against *B. pertussis*. The effect of this combination on protection in the upper respiratory tract was not investigated.

A novel TLR-agonist combination was recently tested as adjuvant for aPV and provided sustained immunity against mouse nasal colonization for at least 10 months after intranasal administration (30). The novel adjuvant combination called LP-GMP comprised the *B. pertussis*-derived lipopeptide LP1569, a TLR-2 agonist, and c-di-GMP, an agonist for the intracellular receptor stimulator of interferon genes. This molecule can synergize with TLR-agonists to enhance immune responses to nasally delivered antigens. c-di-GMP and LP1569 synergistically induced the production of IFN-γ, IL-12 and IL-23, the latter of which activated and expanded Th17 cells. When combined with FHA, recombinant PT and PRN, intranasal immunization with LP-GMP induced potent Th17 T_RM_ cells in the nasal tissue.

##### Mucosal Particulate Formulations

The use of particulate adjuvants has been successful in inducing increased immune responses against *B. pertussis* antigens following oral immunization in mice. Brownlie et al. have shown that liposomes loaded with outer membrane proteins can be used to enhance specific antibody responses to *B. pertussis* that were long-lasting after oral or nasal immunization (31), indicating that liposome loading of outer membrane proteins had adjuvant effects.

The use of polymers, such as polyphosphazenes, as the basis of new adjuvants has also been explored. Polyphosphazenes are versatile organic-inorganic polymers that form microparticles. Intranasal immunization of mice with detoxified PT and poly[di(sodium carboxylatophenoxy)phosphazene] (PCPP) elicited balanced Th1 and Th2 immune responses (32). It enhanced specific systemic and mucosal antibody responses compared to PT alone or PT adjuvanted with the TLR-agonist CpG ODN. PCPP provided as much protection against lung colonization as CpG ODN when formulated with detoxified PT. The PCPP formulation induced long-term systemic, lung and nasal memory B cell responses, whereas memory B-cell responses to CpG ODN-adjuvanted detoxified PT waned quickly. Furthermore, unlike CTB- or LTB-based adjuvants, intranasal administration of PCPP did not direct co-administered antigens into olfactory tissue.

Encapsulation of *B. pertussis* antigens in biodegradable poly-lactide-co-glycolide (PLG) microspheres was similarly evaluated for its adjuvanticity (33). Conway et al. showed that protection could be generated with three oral doses of 100 µg detoxified PT and FHA encapsulated in PLG, which was significantly greater than oral vaccination of the same antigens in a soluble form (34). However, they did not study the adjuvant effect of PLG nanoparticles when injected nasally. These results were in line with those obtained in earlier studies, in which oral administration of fimbriae encapsulated in PLG microsphere elicited both systemic and local production of specific antibodies (35). It also provided protection against colonization 6 weeks after immunization. More recently, *B. pertussis* antigens were trapped into N-trimethyl chitosan (TMC) nanoparticles to enhance the access of antigens administered through the nasal route to sub-epithelial lymphoid tissues (36). The electrostatic interaction between positively charged TMC nanoparticles and anionic glycoproteins present in the mucus layer appeared to increase the residence time of the antigens, which resulted in increased antigen uptake by M cells in the nasal epithelium and subsequent transfer to the sub-epithelial immune cells. Additionally, TMC opens the intercellular tight junctions, thereby facilitating paracellular transport of antigens and increasing antigen absorption. Several studies have demonstrated that chitosan potentiates serum and mucosal immune responses to nasally administered FHA and PT in mice (37, 81). Jabbal-Gill et al. showed that FHA and genetically detoxified PT encapsulated into chitosan induced serum IgG and mucosal IgA after intranasal administration, which were considerably higher than when the antigens were delivered nasally without chitosan (37). Najminejad et al. more recently compared intranasal administration of TMC-encapsulated FHA and detoxified PT with the same antigens formulated in alum given parenterally. They found that both immunizations induced comparable serum antibodies (36). However, in contrast to alum-adjuvanted antigens, nasal immunization with TMC-encapsulated antigens elicited substantial secretion of mucosal IgA and induced predominantly Th1 and Th17 cytokines. Although TMC-loaded antigens are promising vaccine candidates, the protection they confer against *B. pertussis* infection remains to be evaluated.

#### Outer Membrane Vesicles

Most recently, OMV have been proposed as mucosal vaccines against pertussis. OMV are obtained from membrane fractions of *B. pertussis* in which antigens are present in their native conformation, together with membrane-associated PAMPs acting as immunostimulatory molecules. This approach may be less costly than other antigen-controlled-release systems, which may be especially attractive for low- and middle-income countries. Hozbor et al. were among the first to suggest that *B. pertussis* OMV may be attractive pertussis vaccine candidates, as they carry a variety of protective antigens (81). The composition of the *B. pertussis* OMV has been characterized in detail and identified 43 proteins (38), including the virulence factors PT, PRN, fimbriae, FHA and adenylate cyclase toxin (ACT), known to be a strong mucosal adjuvant (82). Intranasal vaccination with the OMV induced the innate immune markers TNF-α, IL-6 and CCL20 and cleared the challenge bacteria as rapidly as intranasal vaccination with wPV (38). Systemic and local, pulmonary and nasal, IgA were substantially increased, as were pulmonary Th1- and Th17-related cytokines after intranasal OMV administration (39). Local responses were not induced when the OMV were delivered subcutaneously. In contrast, comparable systemic responses were induced by either vaccination route. However, only intranasal immunization prevented colonization in both the lungs and nasal lavages.

*B. pertussis* OMV contain endotoxic LOS, which may induce adverse events following immunization. However, LOS toxicity is mainly a concern for parenterally administered pertussis vaccines, while LOS is less toxic when the vaccines are administered mucosally. Nevertheless, in order to decrease LOS toxicity, OMV were subsequently prepared from a recombinant *B. pertussis* strain that expresses the *pagL* gene from *Bordetella bronchiseptica* (40). The product of this gene hydrolyzes the ester bond at position 3 of lipid A. This modification, resulted in decreased toxicity, as shown in a mouse weight-gain test, and decreased lung inflammation after nasal administration. Combined with tetanus and diphtheria toxoids it was safer than wPV, and after intranasal vaccination, the OMV of the recombinant strain was as effective as the original OMV in protection against lung colonization by *B. pertussis*.

Pulmonary vaccination with OMV using a microspray aerosolizer has also been evaluated and was found to lead to improved protection over subcutaneous immunization (41). It induced mucosal IgA and Th17-type responses, as well as systemic IgG, memory B cells and Th17 cells, while only systemic responses were induced by subcutaneous administration of the OMV. However, pulmonary immunization with these OMV did not induce appreciable levels of anti-PT and anti-FHA antibodies.

*In vitro* experiments with lung epithelial cells have revealed that *B. pertussis* OMV strongly adhere to these cells (83), which may be an important property for the induction of strong immune responses upon pulmonary immunization. Optimal adherence was obtained when OMV were prepared from organisms grown in Bvg^+^ conditions in which all know virulence factors, including the aPV vaccine antigens, are produced. This line of work has also resulted in the identification of new factors for bacterial adherence to pulmonary epithelial cells (83). Immunization with OMV vaccines prevented adherence of challenge bacteria to lung epithelial cells and thereby may provide protection against bacterial colonization. The importance of growing the OMV-producing bacteria in Bvg^+^ conditions for effective vaccine production was recently confirmed by comparing OMV prepared from Bvg^+^ bacteria with those prepared from Bvg^-^ bacteria (84). Only the former were able to induce protective immunity against lung infection. In order to stabilize OMV vaccines a thermostable spray dried formulation was developed and found to induce strong mucosal immune responses and to be highly effective in clearing challenge infection in lungs, trachea and nasal lavages after pulmonary immunization (42).

The higher the number of immunogens in the vaccine formulation the lower is the risk of generating vaccine escape *B. pertussis* variants through the deletion or inactivation of vaccine antigen genes. In that regard, OMV vaccines are a promising alternative to current aPV which have imposed selective pressure, as documented by the emergence of isolates lacking PRN (85), one of the prime antigens in many current aPV.

### Replicating Vaccines

Historically, live attenuated replicating vaccines have provided the most effective protection against microbial infection and disease. These vaccines often elicit protective immunity of long duration. By contrast, immunity induced by inactivated or subunit vaccines is generally of more limited duration.

#### Recombinant Vaccines

Attenuated food-borne pathogens or micro-organisms generally regarded as safe (GRAS) have been genetically modified to express *B. pertussis* antigens for oral immunization against pertussis. Attenuated oral *Salmonella* vaccines, that lack the *aroA* gene encoding a synthase crucial for the production of aromatic amino acids, have been used to deliver antigens and to elicit immune responses to both *Salmonella* and heterologous antigens. These mutants are unable to multiply extensively or cause disease in the host, but establish a self-limiting, subclinical infection and can be detected in tissues, such as the liver and spleen. Oral inoculation of mice with a live attenuated strain of *Salmonella dublin* expressing a portion of the *B. pertussis fhaB* gene induced IgA responses to FHA in sera and gut washings (43). However, the antibody responses were not long-lived in animals immunized once. *Salmonella* producing heterologous antigens are also able to induce cell-mediated immunity. A strong anti-PRN proliferative response was observed in murine splenocytes following a single oral dose of the *Salmonella typhimurium ΔaroA ΔaroD* vaccine strain producing PRN (44). This strain induced a Th1 type response against PRN, whereas no antibody response to PRN could be detected following oral immunization. Augmenting the expression of PRN by the use of a strong inducible promoter only slightly enhanced humoral responses. Only subcutaneous boosting with PRN yielded detectable IgG2 in mouse sera. However, in an earlier study, Guzman et al. have shown that oral immunization of mice with live *Salmonella typhimurium ΔaroA* or an invasive *E. coli* strain producing FHA induced serum IgG, as well as IgA and IgG responses in the lungs (45). Similar results were obtained by using *S. thyphimurium ΔaroA* and invasive *E. coli* strains producing the S1 subunit of PT (46). Protection against virulent challenge was not assessed in these studies. Subsequently, a *S. thyphimurium ΔaroA* strain producing all five PT subunits also induced significant IgG specific to PT in mouse serum upon oral administration (47) but did not protect the mice from challenge using an intracerebral challenge model. However, while the intracerebral challenge model is widely used to assess vaccine potency of injectable wPV, it is not an appropriate model to evaluate mucosal vaccines against pertussis.

The oral typhoid vaccine *Salmonella typhi* CVD 908 has also been used as a mucosal delivery system for *B. pertussis* antigens (48). Mice immunized intranasally with several doses of a *S. typhi* CVD 908 strain producing a hybrid protein composed of the PT S1 subunit and tetanus toxin fragment C produced detectable antibodies to these toxins, which were, however, lower than those elicited by parenteral immunization with crude extracts of the recombinant *S. typhi* CVD strain. Although the antibodies to PT neutralized PT activity *in vitro*, protection against lung or nasal colonization was not assessed in this study.

In addition to attenuated *Salmonella* strains, the live attenuated *Vibrio cholerae* vaccine strain IEM101 has also been evaluated as a mucosal delivery system for *B. pertussis* antigens (49). Four intranasal administrations of a recombinant IEM101 derivative secreting the *B. pertussis* tracheal colonization factor were found to reduce the bacterial load in the trachea, but not in the lungs upon challenge infection with *B. pertussis*, although no specific antibodies could be detected in sera and mucosal washes.

LAB, such as lactococci and lactobacilli have also been engineered to produce *B. pertussis* antigens. LAB are classified as GRAS organisms and often used as oral probiotics. Therefore, they are considered as safe mucosal delivery vehicles. Torkashvand et al. constructed a recombinant a *L. lactis* strain that produces and secretes a chimeric protein composed of the N-terminally truncated S1 subunit of PT and an immunodominant domain of FHA (50). Oral or nasal immunization with this recombinant strain induced specific serum IgG and specific IgA in the lungs of the vaccinated mice, as well as Th1-type responses in the spleen. Oral immunization also increased IgA levels in fecal extracts, while nasal immunization elicited better IFN-γ responses in antigen-stimulated splenocytes. No information on a potential protective effect of mucosal immunization with recombinant LAB against infection by *B. pertussis* is yet available.

Another GRAS micro-organism is the commensal oral bacterium *Streptococcus gordonii*. In this carrier system the S1 subunit of PT was surface exposed as a chimeric protein fused to the surface protein SpaP (51), and oral administration of the recombinant strain induced detectable levels of anti-PT IgA in the saliva of mice. The S1-SpaP hybrid protein was also produced in a *Streptococcus mutans* strain, used as an oral vaccine vector. Oral vaccination with this strain also resulted in mucosal IgA responses and a significant reduction in bacterial burden in the lungs challenged with *B. pertussis*, compared to non-vaccinated mice (52).

#### Live Attenuated *Bordetella* Vaccines

As an alternative to the above-described recombinant live vectors, nasal immunization with live attenuated *Bordetella* itself may combine the effectiveness of a single vaccination by live vaccines with the breath of immune responses to *B. pertussis* antigens, as induced by wPV and OMV. The development of replicating *Bordetella* vaccines obviously necessitates attenuation of the virulent bacteria. Live attenuated *Bordetella* vaccines were first developed against *Bordetella bronchiseptica* to protect dogs against kennel cough (86). These live attenuated nasal vaccines have been marketed, but the mechanism of attenuation has remained elusive. An attenuated strain lacking ACT and the type III secretion system has more recently been developed (53). The strain was shown to be safe in mice at high doses, even in TLR4-deficient and in TNF-α-deficient mice, and prevented bacterial colonization of the upper and lower respiratory tract against virulent *B. bronchiseptica*. It also provided a certain level of cross-protection against infection with *B. pertussis* in the lower respiratory tract. Although this strain has not yet been evaluated beyond mouse models, two different *B. bronchiseptica* strain lacking ACT or lacking both ACT and PRN were found to poorly colonize the noses of neonatal piglets and did not induce mucosal IgA after nasal administration, in contrast to nasal infection by virulent *B. bronchiseptica* (87).

An attenuated *B. bronchiseptica* strain lacking the antigens homologous to those included in the aPV currently in use, in addition to ACT and the surface protein BipA, efficiently colonized the upper respiratory tract, but not the lower respiratory tract of mice (54). Inflammation and lung injury were reduced when the strain was compared to the virulent parent strain. Interestingly, while serum antibody responses were also dampened in comparison with infection by the parental strain, a single intranasal administration of the attenuated strain conferred significant protection against infection by both *B. bronchiseptica* and *B. pertussis*.

Several attenuated strains of *B. pertussis* have also been tested as nasal vaccine candidates. The first attenuated *B. pertussis* strain is an *aroA* mutant (55). This strain is defective in lung colonization in outbred mice challenged by aerosol. Three aerosol vaccinations with the live *B. pertussis aroA* mutant were found to protect mice against lung colonization by the virulent parental *B. pertussis* strain. Serum IgG and IgA were induced early after challenge and persisted for up to 36 days post infection. Another mutant in the aromatic amino acid biosynthesis pathway is the *aroQ* mutant, which was shown to persist somewhat longer in the lungs of mice than the *aroA* mutant (56). This led to antibody and cytokine responses to *B. pertussis* antigens, as well as protection against lung colonization after a single, yet high dose delivered intranasally.

A mutant lacking the *ptx* gene and referred as to BPRA also conferred protection against lung colonization after a single intranasal administration, even when administered at an approximately 100-fold lower dose than the *aroQ* mutant (57). BPRA was able to replicate in the mouse respiratory tract, yet it is strongly attenuated, as demonstrated by the absence of systemic effects, such as leukocytosis in BPRA-infected mice, and reduced inflammation of the airways compared to mice infected with the virulent parental strain. In addition to inducing systemic immune responses, BPRA also induced antigen-specific IgG and IgA at distal mucosal sites, such as the genital tract of female mice after intranasal administration (58).

To further attenuate BPRA, the gene coding for the dermonecrotic toxin gene was deleted, and the tracheal cytotoxin release was reduced by at least 99% *via* the overexpression of the *E. coli ampG* gene. In addition, the *ptx* gene was re-introduced with codon substitutions that genetically inactivated PT (59). The resulting strain was named BPZE1 and was highly attenuated but retained the ability to colonize the mouse respiratory tract and to provide protection against colonization in the lungs of both adult and infant mice upon challenge with the virulent parental strain. Intranasal immunization with doses as low as 10^3^ bacteria was sufficient to elicit protective immune responses (60). Specific antibodies to *B. pertussis* antigens, as well as IFN-γ responses were induced. In mice the protective immune responses induced by BPZE1 were long-lasting, for at least up to 1 year after immunization (61, 62). BPZE1 showed significantly higher efficacy to protect infant mice against *B. pertussis* infection than two administrations of aPV. Adoptive transfer experiments of serum and spleen cells from BPZE1-immunized mice demonstrated that both cell-mediated and humoral immune responses were involved in protection induced by BPZE1 (63). Importantly, intranasal administration of BPZE1 also induced an IL-17-dependent secretory IgA-mediated mechanism of protection against nasopharyngeal colonization by *B. pertussis* (64). The substantial induction of Th17 T_RM_ cells in the nose of immunized mice is likely to account for the long-lasting protection conferred by BPZE1. In a heterologous prime/boost study in which infant or neonatal mice were primed by an intranasal dose of BPZE1 and then boosted with aPV, the BPZE1-induced Th1/Th17 responses were maintained after the aP booster (65), while priming with aPV shifted the immune responses to a Th2 profile. The vaccine strain showed a good safety profile, including in immunocompromised animals (66, 67). In addition to protection in mice, BPZE1 was also shown to be safe and protective against pertussis disease and infection by *B. pertussis* in non-human primates (68). The attenuating genetic changes in BPZE1 were shown to be very stable, as evaluated after extensive *in vitro* and *in vivo* passaging (88), and very recently, a lyophilized BPZE1 vaccine formulation was developed and shown to be biologically stable for at least up to 2 years, even when stored at room temperature (89). BPZE1 is now in clinical development and has successfully completed 2 phase I dose-escalating trials, which showed that the vaccine is safe and immunogenic in adults (69, 70). Human vaccination with BPZE1 was also shown to induce B-cell (71) and T cell responses to *B. pertussis* antigens, as well as a broad antibody repertoire (72). Phase 2 trials are close to completion and are designed to provide first proof of concept that the vaccine is able to prevent subsequent infection by *B. pertussis* [ClinicalTrials.gov Identifier: NCT03942406].

All clinical studies with BPZE1 have so far been performed with young adult volunteers, and, while no safety signal has been observed in these studies, it remains to be seen whether the vaccine is safe enough in newborns for large-scale vaccination, the most vulnerable population for severe pertussis. In addition, there are yet no data available on the immunogenicity of live attenuated pertussis vaccines in neonates. However, studies have shown that infants as young as a few weeks of age naturally infected with *B. pertussis* are able to mount very strong immune responses to *B. pertussis* antigens (90).

While waiting for results from extensive safety and immunogenicity data in neonates, BPZE1 may be an attractive vaccine candidate as a stand-alone booster vaccine for adolescents and adults. However, the initial clinical studies have suggested that the take of this vaccine may be hampered by pre-existing antibodies to *B. pertussis* antigens, especially to PRN, which may be elicited through vaccination (69, 70). Therefore, a PRN-deficient BPZE1 derivative has recently been constructed and, in the context of vaccine-induced pre-existing antibodies, was found to colonize the respiratory tract of mice better than the original strain (73). Yet, both vaccines protected mice equally well against colonization by both PRN-producing and PRN-deficient clinical *B. pertussis* isolates. To further improve BPZE1, a derivative producing Fim3 and Fim2 has been constructed and shown to protect against lung colonization by Fim3-only producing clinical *B. pertussis* isolates better than the original BPZE1 strain, which only produces Fim2 (74).

Finally, while live attenuated vaccines in general may also theoretically have safety issues for immune-compromised individuals, extensive pre-clinical studies have shown that BPZE1 is safe, even in severely immune-compromised mice (66, 67). Furthermore, as *B. pertussis* is very sensitive to macrolides, the vaccine strains could very easily be eliminated by antibiotic treatment should unexpected complications occur because of uncontrolled colonization by the live vaccine strains.

## Conclusion

*B. pertussis* is a strictly mucosal pathogen. Dissemination of the organism outside the respiratory tract is extremely rare and has only been observed in severely immune-compromised subjects (91, 92). These conditions can be mimicked in mice by the use of IFN-γ receptor KO mice (93), illustrating the role of cell-mediated immunity to control atypical pertussis disease. While infection with virulent *B. pertussis* causes disseminated disease in IFN-γ receptor KO mice, no disseminated infection was seen in these mice when BPZE1 was given instead of virulent *B. pertussis* (66).

The restriction of *B. pertussis* to the airways, and most often to the upper airways, suggests that the induction of local immunity at these mucosal sites may be instrumental in limiting infection by *B. pertussis* and subsequent transmission of this pathogen (10). Next to measles, pertussis is one of the most contagious air-borne pathogens for humans, with an estimated R_0_ factor of 12-17 in non-vaccinated populations (94). Therefore, effective and long-term control of pertussis requires wide-spread immunization with vaccines that not only protect against disease, as do the currently available vaccines, but also prevent infection and transmission (95). Current vaccines have at best minor effects on infection and transmission (1, 5).

Given the high transmission rates of *B. pertussis* and its strictly mucosal habitat, it is unlikely that improvements of systemic vaccination with current aPV will reach the high bar of interrupting *B. pertussis* circulation in human populations. Considering all available evidence, it is more likely that ultimate control of pertussis relies on the induction of potent and long-lasting immunity in the respiratory tract, which may be achieved through mucosal vaccination.

Nevertheless, compared to the wealth of studies published on systemic vaccination against pertussis, mucosal vaccination has still attracted relatively little attention, although several strategies to induce local immune responses to pertussis have been explored, as summarized in this article (**Table 1**). The vast majority of these studies have so far been limited to murine models. Mucosal vaccination with aPV combined with a variety of adjuvants or formulated in nanoparticles have shown promise in mice. However, these vaccines induce immune responses to only a limited number of antigens, which may lead to the emergence and spread of vaccine escape variants, especially if their potency is high. This is well illustrated by the increase in PRN-deficient clinical isolates since the implementation of PRN-containing aPV (85). The use of OMV with a broad antigen repertoire would be less prone to vaccine escape mutants, but likely requires several vaccine administrations to induce strong local protective immunity.

Live attenuated *Bordetella* strains may be the ideal vaccine candidates, combining the ability to induce broad immunity, as induced by OMV, with, hopefully, the ability to induce protection after a single intranasal administration, as is the case of natural infection with *B. pertussis*. A further advantage of live attenuated vaccines is their ability to induce heterologous protection through the induction of trained innate immunity (96), which has also been demonstrated for live attenuated *B. pertussis* (97). Finally, live attenuated vaccines are less costly than aPV, which may make them particularly attractive for low- and middle-income countries, where today the toll of pertussis is the highest (98).

## Author Contributions

VD wrote the first draft. CL completed the draft. All authors contributed to the article and approved the submitted version.

## Conflict of Interest

CL is listed as inventor of patents concerning the live attenuated BPZE1 vaccine.

The remaining author declares that the research was conducted in the absence of any commercial or financial relationships that could be construed as a potential conflict of interest.
